# Nomophobia and psychological distress in a sample of young adults and adults

**DOI:** 10.1192/j.eurpsy.2024.866

**Published:** 2024-08-27

**Authors:** S. Pinheiro, B. R. Maia

**Affiliations:** ^1^ Universidade Católica Portuguesa; ^2^Universidade Católica Portuguesa, Center for Philosophical and Social Sciences, Braga, Portugal

## Abstract

**Introduction:**

Nomophobia comes from the term “no-mobile-phone phobia” and describes the discomfort, stress, or anxiety caused by the absence of a cell phone or any other virtual communication device in individuals who use these devices frequently. Research, although scarce, points to a statistically significant relationship between nomophobia and psychological distress factors such as anxiety, depression, and stress.

**Objectives:**

To explore the relationship between nomophobia and psychological distress in a sample of young adults and adults

**Methods:**

The sample was composed of 194 Portuguese subjects, aged between 18 and 30 years old (*M* = 22.08, *DP* = 2.89), who sulfilled a sociodeomographic questionnaire, and the Portuguese version of the Nomophobia Questionnaire, and of the Anxiety, Depression, and Stress Scale.

**Results:**

The entire sample showed some type of nomophobic symptomatology, specifically 59.3% (*n* = 106) had moderate nomophobia and 24.2% (*n* = 40) had severe nomophobia. Positive and statistically significant correlations, with strong magnitude, were found between nomophobia and anxiety (.46**), depression (.58**), and stress (.50**) subescales. Females presented significantly higher nomophobia levels (*Md* = 109.35) compared to males (*Md* = 71.66), *U* = 2480.50, *p* <.001, with an hight effect size (*d* =.69). A significant and negative correlation was found between nomophobia and age (.-.18*).

**Conclusions:**

Nomophobia was present in all the sample, and it is related to psychological distress. Females and younger subjects presented higher nomophobia levels. Further studies are needed to clarify their etiology, but some preventive and remediative actions need to be developed in order to minimize its emergence and their negative psychological impact.

**Disclosure of Interest:**

None Declared

